# THE ROLE OF MANAGED CARE IN HOME- AND COMMUNITY-BASED SERVICES SPENDING

**DOI:** 10.1093/geroni/igae098.2260

**Published:** 2024-12-31

**Authors:** Patrick Arp, Lindsay White, Ellen Meara, Ari Ne’eman, Norma Coe

**Affiliations:** University of Pennsylvania, Philadelphia, Pennsylvania, United States; University of Pennsylvania, Philadelphia, Pennsylvania, United States; Harvard University, Boston, Massachusetts, United States; Harvard University, Boston, Massachusetts, United States; University of Pennsylvania, Philadelphia, Pennsylvania, United States

## Abstract

The provision and management of Medicaid-financed long-term services and supports (LTSS) has changed dramatically over the last 25 years, including a substantial expansion of both home and community-based services (HCBS) and managed care programs. However, evidence on the costs of these services and drivers of costs is limited. The objective of this study is to describe LTSS and HCBS spending across states, over time, and to examine the impact of managed care on spending. We used a difference-in-difference approach, accounting for the staggered timing of managed care implementation, to analyze data from annual Medicaid LTSS reports and think tank reports from the year 2000 and onward. We find that, after adjusting for inflation, national LTSS spending increased from approximately $410 per capita in 2000 to $570 per capita in 2021; HCBS spending more than tripled during this period. Nineteen states introduced managed care into their LTSS programs between 2000 and 2021 (seven states had implemented programs prior to 2000). States that used managed care to provide some of their Medicaid LTSS demonstrated lower overall LTSS spending post-implementation, but no differences were observed in HCBS spending. However, uncertain fidelity of the data, particularly among states implementing managed LTSS programs, leads to questions of whether these differential trends are due to differences in reporting or changes in the efficiency of care delivery. Our findings highlight the need for increased accuracy and transparency in spending data to facilitate studies that will produce the evidence base for state and federal Medicaid policy makers.

